# Supplementary material for: Cross-ancestry genetic investigation of schizophrenia, cannabis use disorder, and tobacco smoking
Source: Neuropsychopharmacology. 2024 Jun 21;49(11):1655–65. doi: 10.1038/s41386-024-01886-3 (PMC11399264; doi:10.1038/s41386-024-01886-3)
Supplement: Supplementary file 1 — Supplementary Methods [file 41386_2024_1886_MOESM1_ESM.docx]

**Supplement Methods for “Cross-ancestry genetic investigation of schizophrenia, cannabis use disorder, and tobacco smoking”**

*Genome-wide summary statistics*

- *Ever-smoked tobacco regularly (Smk):* We selected this phenotype over others reflecting smoking quantity (cigarettes per day) or dependence because it had the largest sample size and the most genome-wide significant loci of any tobacco-related GWAS and shows considerable overlap with other nicotine use traits; thus, it seems likely that the genetics of Smk would be inclusive of most genetic factors related to tobacco involvement.

We also used genome-wide summary statistics for attention deficit hyperactivity disorder (ADHD), bipolar disorder, depression, post-traumatic stress disorder (PTSD), educational attainment, executive function, risk-taking, and the Townsend Deprivation Index (TDI) for follow-up analyses:

- *Attention deficit hyperactivity disorder (ADHD)*: We included a GWAS of ADHD^1^ in European ancestry individuals (N_cases_ = 20,183; N_controls_ = 35,191).
- *Bipolar disorder*: We used data from a GWAS of 41,917 bipolar disorder cases and 371,549 controls of European ancestry^2^.
- *Depression*: We included a GWAS of depression^3^ in European ancestry individuals that meta-analyzed data from the MVP, the PGC, and the UK Biobank (N_cases_ = 254,566; N_controls_ = 495,848).
- *Post-traumatic stress disorder (PTSD)*: We used data from a GWAS of PTSD diagnosis^4^ in individuals of European ancestry (N_cases_ = 23,212; N_controls_ = 151,447).
- *Educational attainment:* We used data from a GWAS of educational attainment^5^ in individuals of European ancestry (N = 766,345).
- *Executive function:* We used summary statistics from a GWAS of executive function in the European ancestry subset of the UK Biobank^6^ (2023; N = 427,037).
- *Risk-taking:* We used data from a GWAS ^43^of a single item that queried whether someone was a risk-taker^7^. This GWAS was a meta-analysis of the UK Biobank and 10 replication cohorts (N = 466,571).
- *TDI:* We used summary statistics from the Neale Lab GWAS (<https://www.nealelab.is/uk-biobank>) of the Townsend Deprivation Index (a measure of material deprivation in a region, incorporating data on unemployment, non-car-owning households, non-home-owning households, and household overcrowding) in the European ancestry subset of the UK Biobank (N = 336,798).

*Genome-wide genetic correlation analyses*

We tested whether genetic correlations were significantly different from each other using a block-jackknife method^8,9^. The block-jackknife method is a resampling approach, where the difference between resampling genetic correlations is used to calculate a jackknife standard error. From this standard error a Z-statistic is estimated and used in a two-tailed Z-test to determine if the difference between two genetic correlations is significantly different from zero (i.e., H_0_: r_g_(Scz, Smk) - r_g_(Scz, CanUD) = 0).

*Causal inference analyses*

CAUSE constructs two nested models: a sharing model and causal model. Both models allow for horizontal pleiotropic effects; however, only the causal model includes a causal effect parameter (gamma). CAUSE compares the sharing and causal models to each other, to determine which model best fits the data, by estimating the difference in the expected log pointwise posterior density (ΔELPD). CAUSE then computes a z-score from the ΔELPD that can be compared to a normal distribution to obtain a one-sided p-value, which corresponds to a test of the null hypothesis that the sharing model fits the data at least as well as the causal model. Significant p-values therefore indicate the presence of a causal effect, after accounting for pleiotropy. Following the CAUSE package guidance, the r^2^ threshold was set to 0.01 and the p-value threshold was set to 1e-3 for clumping. Similarly following the package's tutorial, the nuisance parameters were obtained using a random sample of 1 million SNPs.

In our MR analyses, we required SNP instruments to have p-value < 5e-8 and performed LD-based clumping using the default parameters (clumping window = 10,000 kb, clumping r^2^ threshold = 0.001, using 1000 Genomes European ancestry data for LD reference). The smallest number of instruments analyzed was 18 (for CanUD). Exposure and outcome data were harmonized using the harmonise_data() function from the TwoSampleMR package.

*Cross-disorder genome-wide association study meta-analysis*

We used the default parameters for FUMA, with “independent SNPs” defined as those with p < 5e-8 and independent of each other with LD r^2^ < 0.6, and “lead SNPs” as independent SNPs which are strictly independent at a more stringent LD r^2^ < 0.1. Genomic risk loci (defined by LD blocks of independent SNPs) that were 250 kb or closer were merged into a single locus.

*BioVU PheWAS*

Analyses were conducted using the PheWAS version 0.99.5-2 R package. Phecodes were excluded from the analysis if they did not have at least two International Disease Classification codes mapping to a PheWAS disease category (Phecode Map 1.2; <https://phewascatalog.org/phecodes>) and had less than 100 cases. The disease phenotypes included 145 circulatory system, 123 genitourinary, 118 endocrine/metabolic, 125 digestive, 118 neoplasms, 91 musculoskeletal, 85 sense organs, 73 injuries & poisonings, 68 dermatological, 76 respiratory, 69 neurological, 64 mental disorders, 42 infectious diseases, 42 hematopoietic, 34 congenital anomalies, 34 symptoms, and 31 pregnancy complications. The phenome-wide significance threshold was set at a Bonferroni-adjusted threshold of *p* ≤ 3.62e-5.

1. Demontis, D. *et al.* Discovery of the first genome-wide significant risk loci for attention deficit/hyperactivity disorder. *Nat Genet* **51**, 63–75 (2019).

2. Mullins, N. *et al.* Genome-wide association study of more than 40,000 bipolar disorder cases provides new insights into the underlying biology. *Nat Genet* **53**, 817–829 (2021).

3. Levey, D. F. *et al.* Bi-ancestral depression GWAS in the Million Veteran Program and meta-analysis in >1.2 million individuals highlight new therapeutic directions. *Nat Neurosci* **24**, 954–963 (2021).

4. Nievergelt, C. M. *et al.* International meta-analysis of PTSD genome-wide association studies identifies sex- and ancestry-specific genetic risk loci. *Nat Commun* **10**, 4558 (2019).

5. Lee, J. J. *et al.* Gene discovery and polygenic prediction from a genome-wide association study of educational attainment in 1.1 million individuals. *Nat Genet* **50**, 1112–1121 (2018).

6. Hatoum, A. S. *et al.* Genome-wide association study shows that executive functioning is influenced by GABAergic processes and is a neurocognitive genetic correlate of psychiatric disorders. *Biol Psychiatry* **93**, 59–70 (2023).

7. Linnér, R. K. *et al.* Genome-wide association analyses of risk tolerance and risky behaviors in over 1 million individuals identify hundreds of loci and shared genetic influences. *Nat Genet* **51**, 245 (2019).

8. Coleman, J. R. I. *et al.* Genome-wide gene-environment analyses of major depressive disorder and reported lifetime traumatic experiences in UK Biobank. *Mol Psychiatry* **25**, 1430–1446 (2020).

9. Bulik-Sullivan, B. K. *et al.* An atlas of genetic correlations across human diseases and traits. *Nat Genet* **47**, 1236 (2015).
