# Supplementary material for: Cross-ancestry genetic investigation of schizophrenia, cannabis use disorder, and tobacco smoking
Source: Neuropsychopharmacology. 2024 Jun 21;49(11):1655–65. doi: 10.1038/s41386-024-01886-3 (PMC11399264; doi:10.1038/s41386-024-01886-3)
Supplement: Supplementary file 3 — Supplementary Figure 1 [file 41386_2024_1886_MOESM3_ESM.docx]

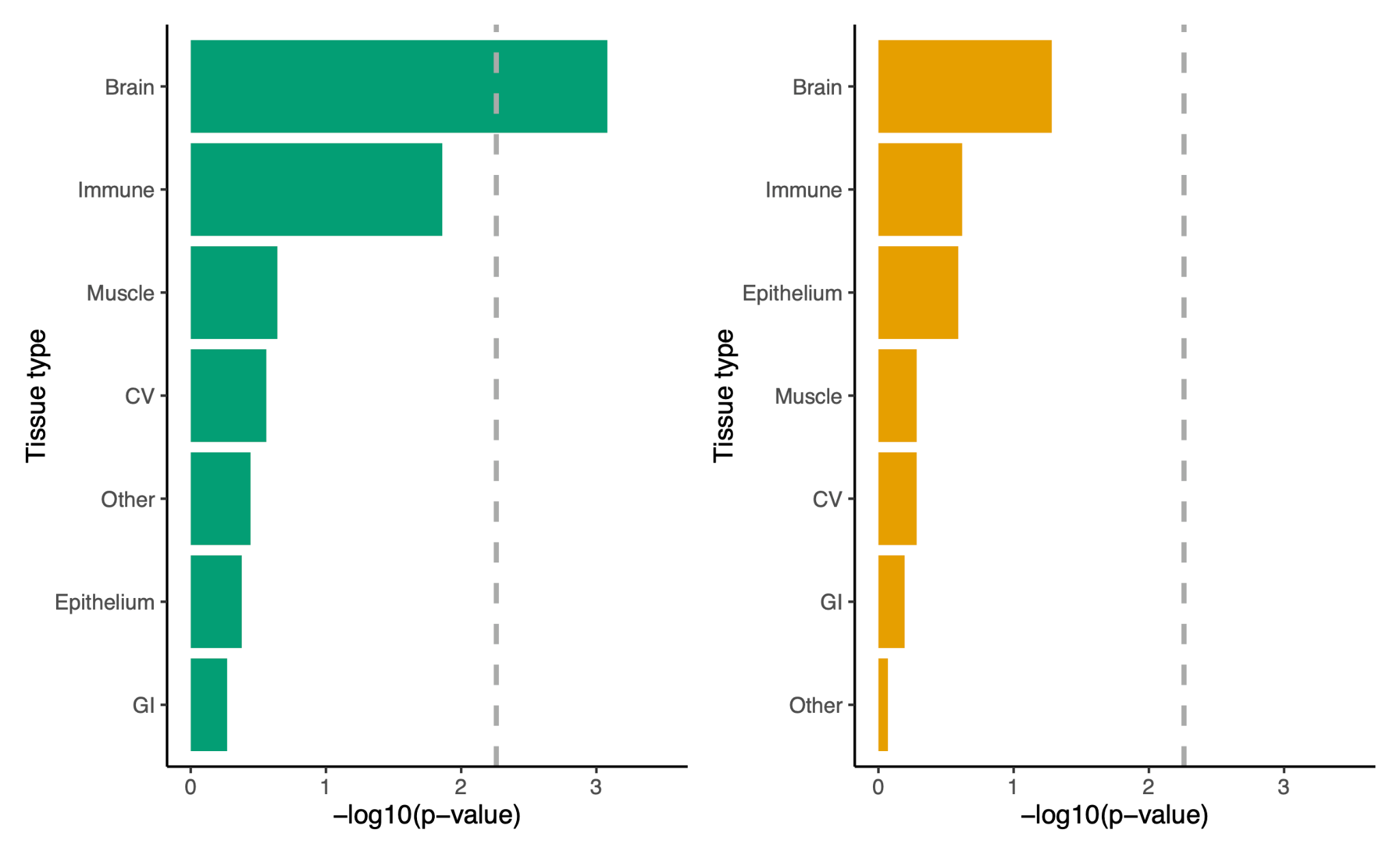


**Supplemental Figure 1. Enrichment of the genetic covariance between cannabis use disorder and schizophrenia (green) and tobacco smoking and schizophrenia (orange) in broad tissue types.** Statistical significance after Bonferroni correction for multiple testing is indicated by the dashed gray line. CV = cardiovascular; GI = gastrointestinal.
